# Lawmakers Order EPA To Clean Up Work Environment

**DOI:** 10.1100/tsw.2000.3

**Published:** 2001-04-04

**Authors:** Nicole Ruediger

## Abstract

Washington, Oct 6 — On Wednesday, members of Congress ordered the Environmental Protection Agency (EPA) to do a “Superfund” cleanup of its own work environment. For 3 hours, lawmakers and the public listened as witnesses testified to patterns of intolerance, discrimination, and retaliation within the EPA — “This agency is run like a 21st century plantation and this has to stop,” said witness and EPA policy analyst Marsha Coleman-Adebayo.

Wednesday's hearing came on the heels of a Department of Occupational Safety and Health Administration (OSHA) ruling Monday stating that it had found "credible evidence" that the EPA had improperly retaliated against Rosemarie Russo, Director of the EPA Office of Research and Development (ORD) in Athens, Georgia after she provided testimony to the House Committee on Science for an EPA oversight hearing and for allowing one of her colleagues, David Lewis, to publish a paper in the journal *Nature* which called into question EPA risk assessments.

"It is entirely inappropriate for any agency that calls itself a scientific laboratory to engage in any behavior of that sort," Rep. Vernon Ehlers (R-MI) said to EPA Administrator Carol Browner.

Browner countered the testimony by citing figures showing that the numbers of minorities in the Senior Executive Service at the EPA have more than tripled and the number of women employed by the EPA has increased by 50%. Browner also said that the EPA has "made great strides" in improving the quality of its science. However, Rep. F. James Sensenbrenner Jr. (R-WI), chairman of the House Committee on Science concluded "all is not well at the EPA."

"I think," said Sensenbrenner, "that since this administration is going out of office in a little over 3 ½ months, it is important that you spend your personal time between now and January 20 cleaning up this mess so that the new President and whomever he appoints as EPA administrator does not get a can of garbage to start out the new administration."

—Nicole Ruediger

Lewis, D.L. et al. (1999) Influence of environmental changes on degradation of chiral pollutants in soils. *Nature*. **401**, 898-901.

Previously posted in NewsLab, TheScientificWorld.
© 2000 TheScientificWorld, Inc.
